# Molecular Mechanism of Resveratrol’s Lipid Membrane Protection

**DOI:** 10.1038/s41598-017-18943-1

**Published:** 2018-01-25

**Authors:** Qinqin Fei, David Kent, Wesley M. Botello-Smith, Fariah Nur, Saadia Nur, Abdelaziz Alsamarah, Payal Chatterjee, Maria Lambros, Yun Luo

**Affiliations:** Western University of Health Sciences, College of pharmacy, Pomona, CA 91766 USA

## Abstract

Resveratrol, a natural compound found in red wine and various vegetables, has drawn increasing interest due to its reported benefit in cardiovascular protection, neurodegenerative disorders, and cancer therapy. The mechanism by which resveratrol exerts such pleiotropic effects remains unclear. It remains as one of the most discussed polyphenol compounds in the debating "French Paradox". In this study, using molecular dynamics simulations of dipalmitoyl phosphatidylcholine (DPPC) bilayer with resveratrol, we generated a free energy map of resveratrol’s location and orientation of inside the lipid bilayer. We found that resveratrol increases the surface area per lipid and decreases membrane thickness, which is the opposite effect of the well-studied cholesterol on liquid phase DPPC. Most importantly, based on the simulation observation that resveratrol has a high probability of forming hydrogen bonds with *sn-1* and *sn-2* ester groups, we discovered a new mechanism using experimental approach, in which resveratrol protects both *sn-1* and *sn-2* ester bonds of DPPC and distearoyl phosphatidylcholine (DSPC) from phospholipase A1 (PLA1) and phospholipase A2 (PLA2) cleavage. Our study elucidates the new molecular mechanism of potential health benefits of resveratrol and possibly other similar polyphenols and provides a new paradigm for drug design based on resveratrol and its analogs.

## Introduction

**Figure 1:**
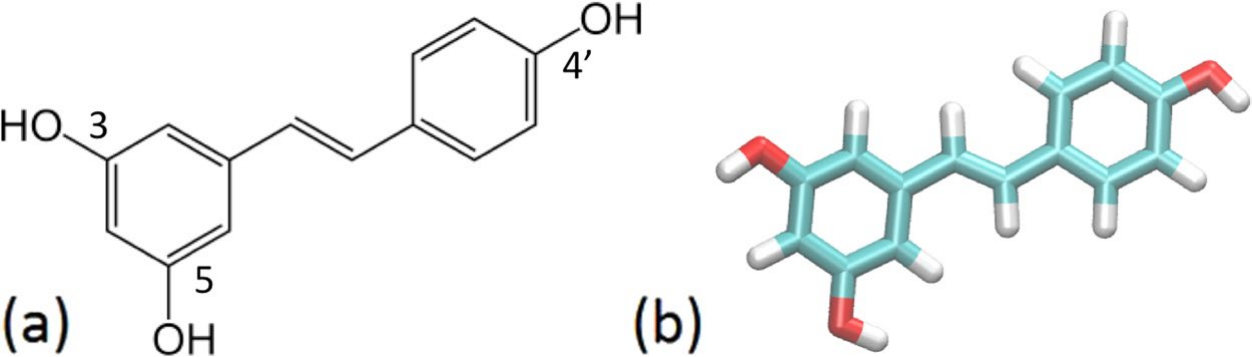
The two dimensional (**a**), and three-dimensional structure (**b**) of a trans-resveratrol molecule.

Resveratrol (Fig. 1) is a natural polyphenol found in red wine, and in several plants such as grapes, raspberries, mulberries, blueberries, and peanuts^1^. The “French Paradox”, the epidemiological observation that consumption of red wine is associated with reduced rate of coronary heart disease, (CHD), sheds light on resveratrol as an agent with cardio protective properties. Resveratrol is a well-known scavenger of free radicals^2^_._ It has been implicated as the mitigating factor reducing the risk of CHD in diets loaded with fats and is considered to have several beneficial attributes such as protection of vascular walls, antioxidant and anti-inflammatory effects, and inhibition of LDL oxidation^3^. Despite resveratrol’s wide consumption for its important health benefits in the prevention of oxidative stress and protection against cardiovascular disease, the molecular mechanism of resveratrol’s protection remains unclear^4–6^.

Resveratrol is known to protect cells from inflammation caused by cytokines and NADPH oxidases released in response to phospholipase A2 (PLA2) induction^7–9^. The acyl ester groups of lipid membranes are susceptible to the cleavage by phospholipases. Specifically, PLA2 is known to damage cell membranes by hydrolyzing the *sn*-2 ester bond of phospholipids and releasing a free fatty acid and a lysophospholipid. Lysophospholipids as surfactants can further damage membranes by solubilizing them. Lipoprotein associated PLA2 contributes to oxidative stress, thus increasing the hydrolysis of low-density lipoprotein (LDL) resulting in increased endothelial dysfunction and stiffness^10^. Therefore, people with a high concentration of lipoprotein associated PLA2 are at high risk of cardiovascular disease and stroke^11^. Both PLA2 and lysophospholipids have been implicated in cancer^12,13^. Phospholipase A1 (PLA1) hydrolyzes the *sn-1* ester bond of phospholipids releasing a free fatty acid and a lysophospholipid. While the function of PLA2 has been well studied, the cellular and physiological function of PLA1 has not been clarified yet^14,15^.

Resveratrol has poor water solubility and its partition coefficient in 1,2-dipalmitoyl-*sn*-glycero-3-phosphocholine (DPPC) and 1,2-distearoyl-*sn*-glycero-3-phosphocholine (DSPC) liposomal membranes in the gel phase is 3.07 and 3.11, respectively^16^. This indicates a high affinity for the lipid part of membranes. Several studies in the literature examine the interaction of resveratrol and its location within model lipid membranes using a variety of techniques such as differential scanning calorimetry (DSC), NMR, X-ray, neutron diffraction, ESR, molecular docking, and molecular dynamics simulations^4–6,16–19^. Overall, it is generally agreed that the hydroxyl groups of resveratrol interact with the head groups of phospholipids, although debate regarding the exact position and orientation of resveratrol within the bilayer continues. Some studies show that resveratrol localizes within the polar headgroup of membrane phospholipids^4,19^ and at high concentrations induces chain interdigitation in DPPC membranes^5^. Others state that resveratrol locates within the acyl chains of the bilayer protecting the polyunsaturated acyl chains from peroxidation^16^. Resveratrol was also reported to decrease the fluorescence of probes located in the hydrocarbon core of the membrane^20^. The preferred orientation of resveratrol in the bilayer has been shown almost flat from a molecular docking study^5^, but more tilted in a previous molecular dynamics simulation of its derivatives^17^. The position of resveratrol within the bilayer has also been discussed as concentration dependent^18^. Besides the location, the effects of resveratrol on the structure and fluidity of the lipid bilayer, it has been reported that resveratrol resembles cholesterol action on DMPC membrane and membrane models consisting of a mixture of egg-phosphatidyl choline, cholesterol, and sphingomyelin^6,21,22^.

Inspired by those previous studies, here for the first time, we used extensive molecular dynamics simulation to map the free energy landscape of resveratrol distribution in a well-parameterized DPPC bilayer model. The free energy landscape clearly reveals the most energetically favorable location and orientation of resveratrol molecules in both the liquid phase and transition phase of DPPC bilayer. We further conducted DSC and show that resveratrol interacts with membranes and shifts the phase transition temperature (Tm) of the DPPC and DSPC to lower temperatures. The simulation results strongly suggest that the resveratrol molecules may protect both ester groups of the lipid head group. We then proved this computational hypothesis using enzyme assays, and demonstrated, for the first time, that resveratrol protects membranes from both PLA2 and PLA1 enzymatic hydrolytic attack of the *sn-2* or the *sn-1* ester groups of the phospholipids. The current study reveals new evidence, from the atomistic level to the cellular level, that resveratrol protects lipid membranes from hydrolysis. Prevention of PLA2 associated cell damage and inflammation can possibly explain the wide range of the health benefit provided by resveratrol intake. More studies are needed to investigate whether this new molecular mechanism may be general for a large variety of naturally occurring polyphenol compounds.

## Results and Discussion

### Resveratrol reduces phase transition temperature in a concentration-dependent manner

**Figure 2:**
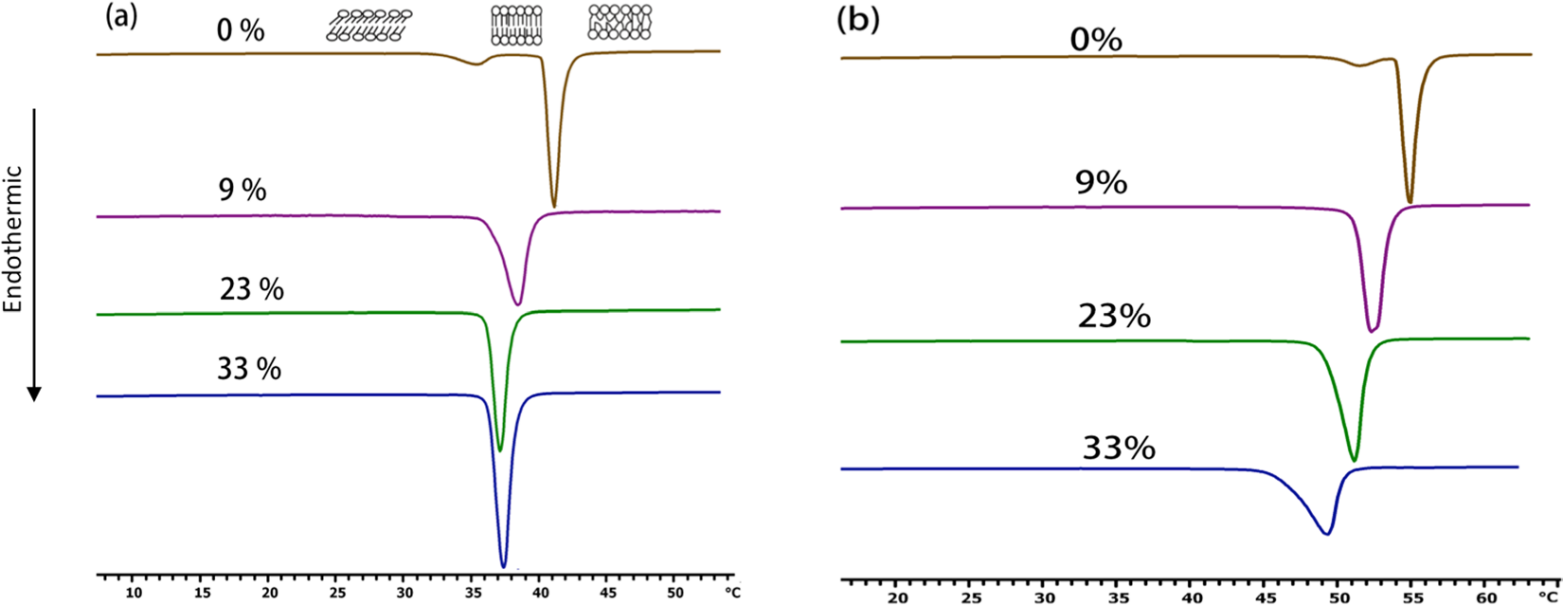
DSC Scans of phospholipid membranes with various amounts of resveratrol (mole %): (**a**) DPPC, (**b**) DSPC.

DSC scans of both DPPC and DSPC membranes in pure condition show a small pre-transition peak at lower temperature accompanying a larger main transition peak at a higher temperature (Fig. 2). The small peak is the pre-transition peak and corresponds to the transition of lipids from Lβ’ phase, where the phospholipids are ordered but tilted with respect to the bilayer normal, to Pβ’ phase, where the lipids are ordered and the bilayer shows ripples. Lipids in either Lβ’ or Pβ’ phase are considered to be in “solid” or “gel” crystalline state. The large peak at the higher temperature represents the main transition, Lα, and corresponds to the transition of lipids from the ‘gel’ to the ‘liquid’ or ‘fluid’ crystalline state. The main transition is a highly cooperative process, meaning that the lipids tend to ‘melt’ in ‘unison’ and the resulting transition peak is narrow. Molecules that interact with phospholipids cause the main transition peak to widen, an indication of loss of the cooperativity between the phospholipids. Resveratrol abolishes the pre-transition of both lipids, reduces the T_m_ of phospholipids in a concentration-dependent manner (Fig. 2). Resveratrol also widens the main transition for both DPPC and DSPC, as shown by the increase of the thickness of the transition peak at half-height (ΔT_1/2_) (Table 1). This indicates loss of cooperativity between the phospholipid molecules. The effect of resveratrol on the melting point of DPPC and DSPC is summarized in Table 1. The reduction in Tm and loss of cooperativity observed here are consistent with literature reports^5,17^. The resveratrol derivative piceid, other resveratrol analogs, and some aromatic compounds, such as fluorene and indeno (1,2,3-ed)pyrene also have similar thermotropic effects on DMPC, DPPC and DSPC as resveratrol^16,23^. However, the membrane thermotropic effect of small aromatic molecules such as local anesthetics which diffuse through membranes is variable and has been used to show that their diffusion through membranes depends on the protonation of the molecule and the state of the membrane^24^. Comparing the thermotropic behavior of resveratrol to that of cholesterol, which markedly reduces the DPPC cooperativity at 30 mole %^25–27^, resveratrol does not reduce the cooperativity of DPPC to the same extent. This is likely due to the fact that cholesterol interacts with the acyl chains more, while resveratrol locates close to the lipid headgroup as we show in our computer simulations and reported by other experiments^5,28^.

**Table 1:** Effect of Resveratrol on the melting point of DPPC* and DSPC*.

Resv. in DPPC (mole %)	Tm (°C)	ΔT_½_ (°C)	Resv. in DSPC (mole %)	Tm (°C)	ΔT_½_ (°C)
0	41.19 ± 0.10	1.00 ± 0.09	**0**	54.95 ± 0.29	1.02 ± 0.12
9	38.94 ± 0.29	1.79 ± 0.12	**9**	52.27 ± 0.27	1.48 ± 0.29
23	37.15 ± 0.30	1.54 ± 0.29	**23**	50.85 ± 0.29	1.65 ± 0.06
33	37.55 ± 0.33	1.47 ± 0.11	**33**	49.50 ± 0.30	2.20 ± 0.18

*Each average value and standard deviation is calculated from three independent measurements (n = 3); Tm is transition temperature; ΔT_1/2_ is the increasing of the width of the transition peak at half-height.

### Effect of resveratrol molecules on DPPC bilayer

**Figure 3:**
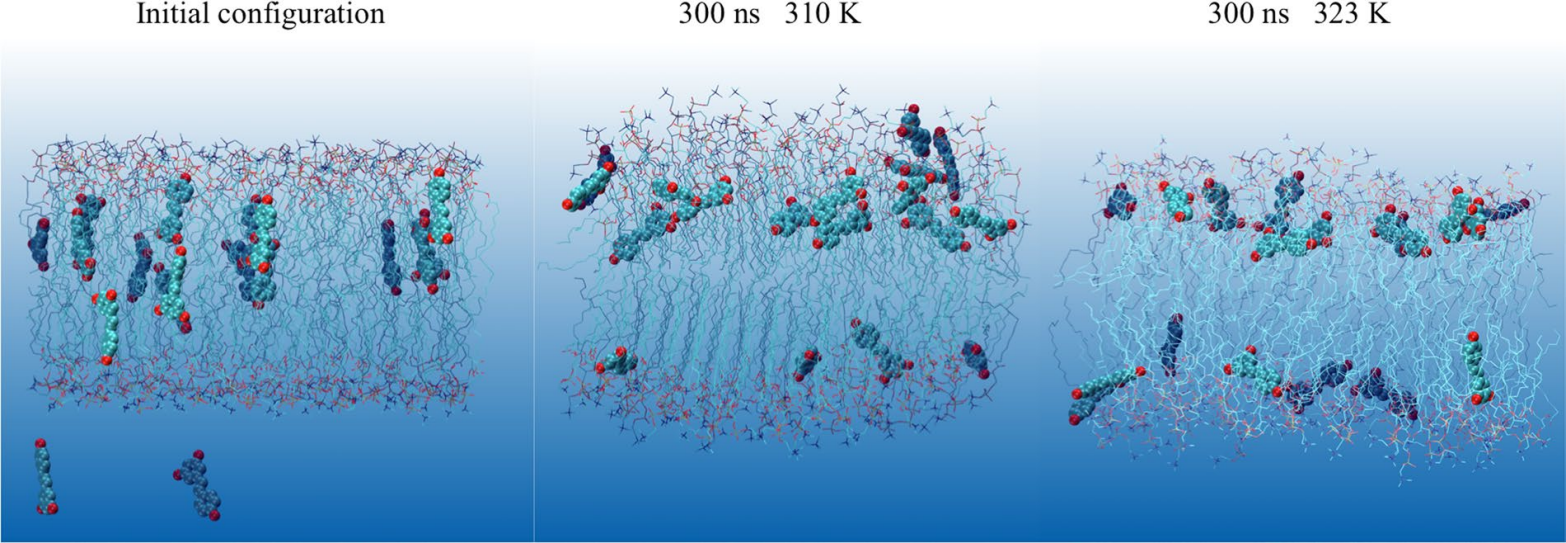
Distribution of resveratrol molecules in DPPC lipid bilayer. First snapshot and last snapshot of the simulated system at 310 K and 323 K. 18 resveratrol molecules are shown in VDW mode with atom color and DPPC lipids are shown in line with atom color: green for carbon, blue for nitrogen, red for oxygen, tan for phosphorus. All hydrogen atoms and water molecules are omitted for clarity.

**Figure 4:**
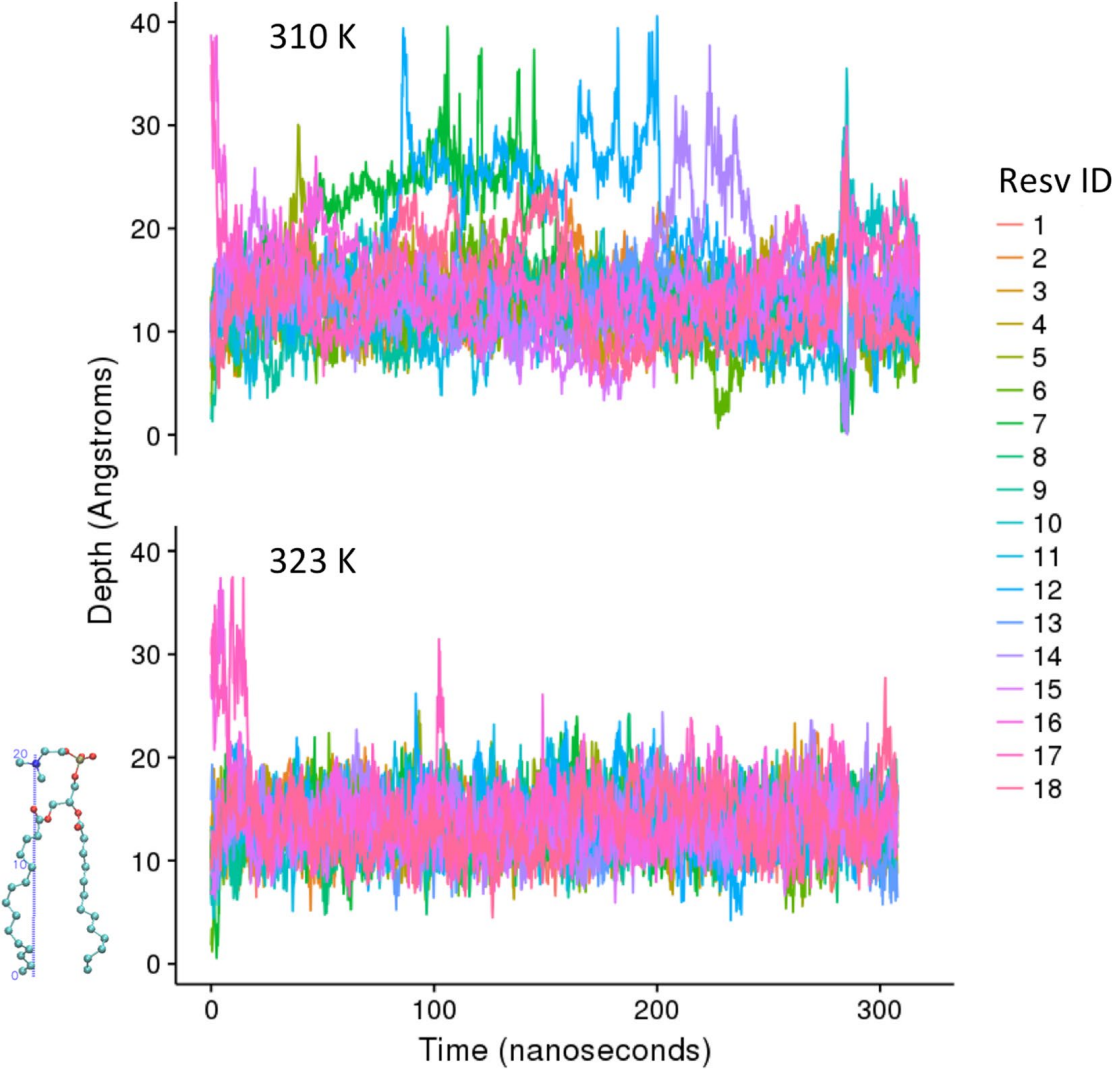
Depth of resveratrol molecules in DPPC lipid bilayer. The distance between the center of mass of 18 resveratrol molecules and bilayer center along z-axis during the 300 ns simulations at two temperatures, 310 K (top) and 323 K (bottom). Each resveratrol is indicated with unique color code. Bilayer center is located at z = 0 Å and the average center of mass of polar head are located at z = 20.5, illustrated on a lipid molecule.

Computational molecular dynamics simulations were performed to evaluate the distribution and interactions of DPPC and *trans*-resveratrol. DPPC bilayer model with CHARMM36 force field has shown good correlation with experimental properties^29,30^. A total of 18 resveratrol molecules were added to a solvated DPPC bilayer system. 16 resveratrol molecules were randomly placed in the DPPC bilayer of 80 DPPC per leaflet, which is equal to the experimental ratio of lipid to resveratrol 10:1. Two additional resveratrol molecules were placed in the bulk solution 15 Å away from the surface of the bilayer (Fig. 3). The overall simulated system contains 10.1% resveratrol. 300 nanoseconds (ns) simulation was conducted at a pressure of 1 bar and at body temperature (310 K or 37 °C) and also at higher temperature 325 K (52 °C). 325 K is the standard temperature used to benchmark DPPC parameters in the liquid phase in comparison with experimental data. Figure 3 shows that all resveratrol molecules diffuse to the head groups of DPPC from bulk or from the bilayer center. The center of mass distance between each resveratrol molecule and bilayer center is shown at Fig. 4. Most of the resveratrol molecules diffuse quickly to the region under the polar group within 20 ns and fluctuate within 10 Å range during 300 ns simulation. It is clear that the binding site of resveratrol inside the DPPC bilayer is shallower (more towards the surface) compared with cholesterol^31,32^. This is due to the distinct feature that cholesterol only has one hydroxyl group, but resveratrol has multiple polar hydroxyl groups on both ends of the molecule. We have shown in the analysis below that there is a high probability that both polar ends of resveratrol form hydrogen bonds with DPPC head groups.

### Resveratrol increases DPPC bilayer surface area and decreases bilayer thickness

**Figure 5:**
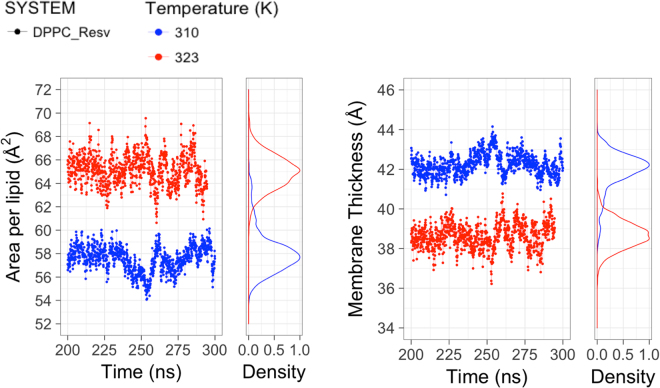
The effect of resveratrol molecules on DPPC lipid surface area and bilayer thickness over last 100 ns simulations at 310 K and 323 K. Left panel shows the time evolution of surface area per lipid and the corresponding distribution for the DPPC simulation with resveratrol. Right panel shows the time evolution of bilayer thickness and the corresponding distribution for the DPPC simulation with resveratrol. The average values are compared with pure DPPC simulations in the Table 2.

In addition to monitoring the distribution of resveratrol molecules inside bilayer, the convergence of the simulations was also determined from the DPPC area per lipid and bilayer thickness. The area per lipid was computed using a triangulation technique to generate a polygon from the coordinates of three atoms in a DPPC molecule (C2-carbon connecting *sn*-2 acyl chain, C31-first carbon on *sn*-1, C21-first carbon on *sn*-2). The bilayer thickness was measured using the distance between the mass density peaks of phosphorus atoms along the membrane normal. Both properties converge within 120 ns and the trajectories from 200 to 300 ns were used to calculate the average values and standard deviations (Fig. 5). At 323 K, the area per DPPC molecule without resveratrol is 62.8 Å^2^ and with 10% resveratrol is 64.9 Å^2^, both corresponds to the liquid crystalline phase. At 310 K, the area per DPPC without resveratrol is 51.7 Å^2^ and with 10% resveratrol is 58.1 Å^2^, both corresponds to the gel to liquid crystalline phase transition. A recent simulation using the same force field also indicates that pure DPPC bilayer at 310 K is between liquid crystalline and condensed phase^33^. Noticeably, the presence of 10% resveratrol shifted the bilayer at 310 K towards the liquid crystalline phase, which is in agreement with our DSC scan data that 9% resveratrol molecules lowered the DPPC transition temperature from 41.19 °C to 38.94 °C (Table 1). Compared with DPPC simulations without resveratrol, the presence of resveratrol increases the bilayer surface and makes the bilayer thinner at both temperatures (Table 2). This is distinct from the effect of cholesterol, which is reported to decrease the area per lipid and increase membrane thickness at liquid phase^34–36^. The opposite effect of resveratrol and cholesterol on liquid phase DPPC bilayer can be explained by the observation that cholesterol interacts more with acyl chain^31^, while resveratrol binds mainly near the headgroup region, having less contact with acyl chain.

**Table 2:** Average values of DPPC lipid area and bilayer thickness with and without resveratrol over last 100 ns simulations.

System	Temperature	Area per lipid (Å^2^)	Bilayer thickness (Å)
DPPC (exp)^a^	323 K	63.0	39.0
DPPC^b^	323 K	62.8 ± 3.0	39.6
DPPC-Resv	323 K	64.9 ± 1.6	38.7 ± 0.6
DPPC	310 K	51.7 ± 0.5	44.9 ± 0.5
DPPC-Resv	310 K	58.1 ± 0.9	41.9 ± 0.9

^***a***^The experimental data of pure DPPC bilayer at 323 K are taken from ref.^37^. ^***b***^Value of pure DPPC system at 323 K is taken from a reported NAMD simulation^29^ with the same C36 force field and same 10–12 Å of the force-based switching range as current simulations. Value of pure DPPC system at 310 K is calculated from 100 ns simulation with the same protocol.

### Resveratrol decreases calculated DPPC deuterium order parameters

**Figure 6:**
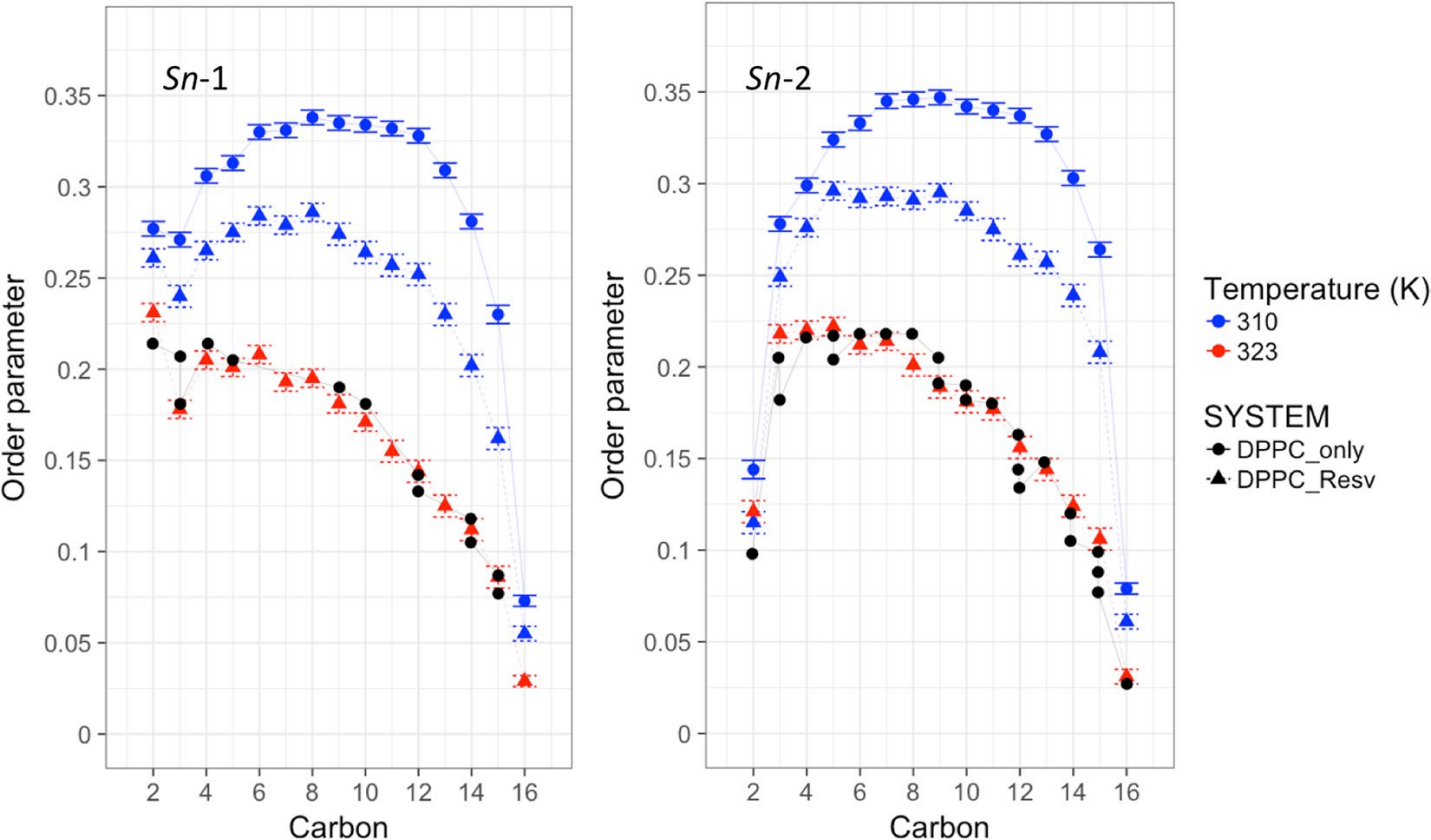
Calculated NMR deuterium order parameters (*S*_CD_) for DPPC bilayer with (dashed line) and without (solid line) resveratrol at two temperatures from simulations. The experimental values at 323 K are taken from refs^38–43^ (black).

Another way to investigate the effect of resveratrol on the lipid bilayer is the deuterium order parameters (*S*_CD_). NMR *S*_CD_ values are a measure of the orientational mobility of the carbon-deuterium bond. Lower *S*_CD_ value usually corresponds to more disordered system bilayer. Here we use the C-H vector to calculated *S*_CD_ values using ${S}_{CD}=-\frac{1}{2}3{\cos }^{2}{\rm{\theta }}-1$, where θ is the instantaneous angle between the C-H bond and the bilayer normal. Figure 6 shows the *S*_CD_ values for *sn*-1 chain and *sn*-2 chain. The definition of *sn*-1 and *sn*-2 chain follows the convention, in which *sn*-2 chain is the tail attached to the oxygen atom of the second carbon of the glycerol group. It is clear that at 310 K, resveratrol greatly decreased the rigidity of the bilayer. The order parameters do not decrease further by resveratrol at 323 K since the bilayer is in liquid phase, as we shown in surface area data above.

### Mapping resveratrol’s location and orientation in DPPC

**Figure 7:**
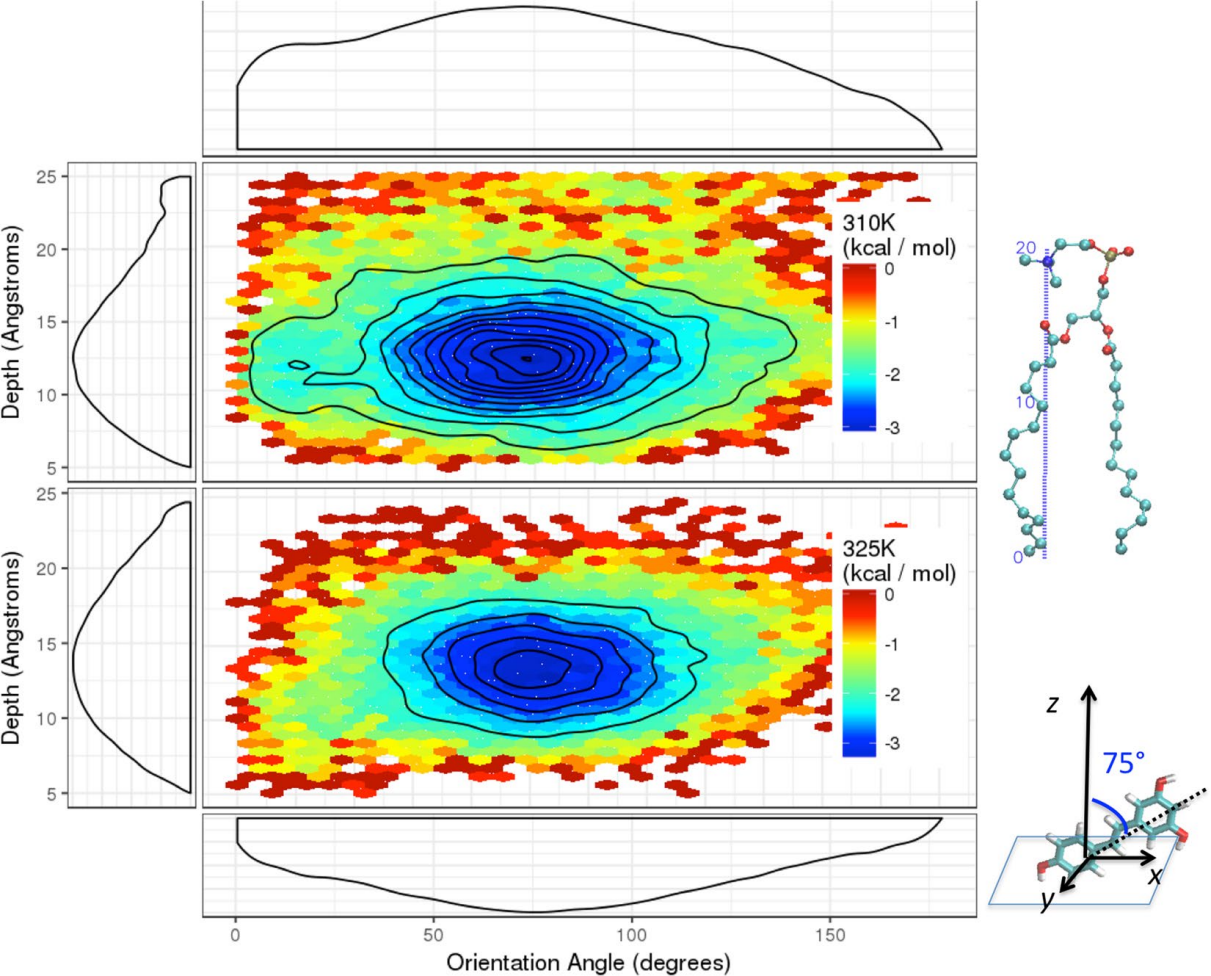
Free energy potential map of resveratrol distribution in the lipid membrane. The *x*-axis indicates the free energy of the angle distribution (illustrated on a resveratrol molecule) and *y*-axis are the free energy along membrane normal (illustrated on a DPPC molecule). The 2D-free energy maps are calculated from the last 100 ns simulation at 310 K and 325 K temperatures. The integrated 1D-free energy profiles of resveratrol’s angle along z-axis are shown at the top and bottom of the maps. The integrated 1D-free energy profiles of resveratrol’s center of mass z-position along membrane normal are shown on the left of the maps.

As indicated from the simulation at 310 K temperature, resveratrol molecules fluctuate throughout the trajectory and occasionally move above the lipid headgroup. It is thus important to analyze the probability distribution rather than looking at individual molecules. We accumulated data from all 18 resveratrol molecules using the snapshots saved from every 2 picoseconds for the last 90 ns, which gives a total 810,000 snapshots (45000 time frames * 18 molecules). We converted the density plot of resveratrol along membrane normal (axis z) and tilt angle of resveratrol along the membrane normal to a two dimensional (2D) free energy contour plot for each temperature (Fig. 7). It is clear that at both temperatures, the location of the center of mass of resveratrol is highly energetically favorable around z = 12 Å, which is located right below the head group and next to the ester groups. This location has been suggested from a steady-state fluorescence quenching experiment^6^. The free energy profile of angular distribution shows much broader distribution than the z location profile. Our data show that it is energetically unfavorable for resveratrol to bind horizontally or vertically near the membrane surface. Instead, resveratrol molecules prefer to be tilted, with the most favorable angle as 75 degrees along membrane normal. The consistency between the two free energy maps further ensures that good convergence of the resveratrol distribution has been obtained in both simulations. It is not surprising that at the higher temperature of 325 K, which is above the DPPC transition temperature, the free energy landscape converges faster and is smoother than the one obtained at lower temperature in both reaction coordinates since the lipid is in the liquid phase. One dimensional free energy profiles along z-position and angle orientation are plotted next to the 2D free energy landscape by integrating out the other dimension.

**Figure 8:**
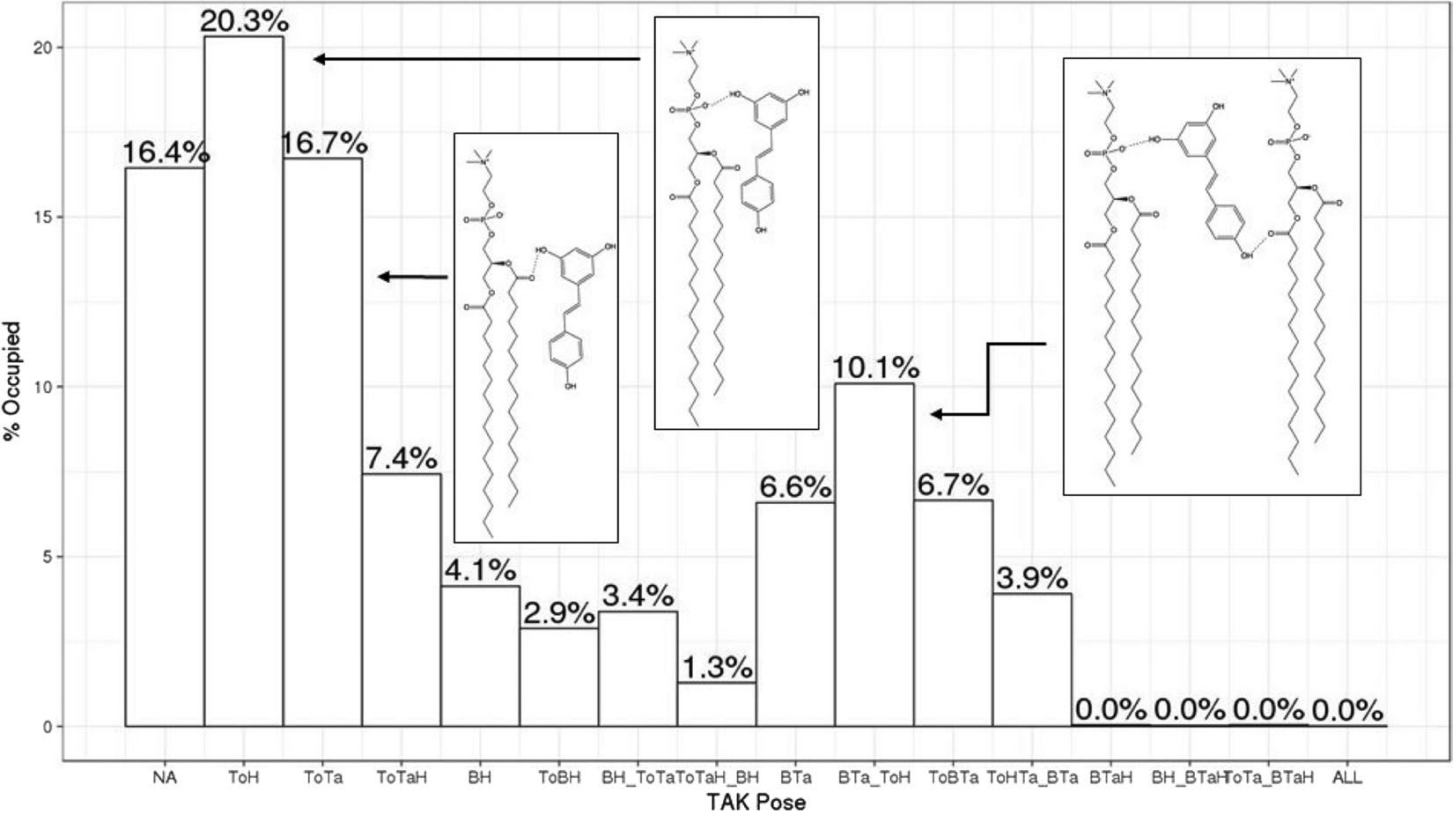
Probability of various types of hydrogen bonds (H-bonds) between resveratrol (Resv) and DPPC. Those that have higher than 10% probability are illustrated with insets. NA: no H-bond; H-bonds between two groups are indicated by the following four-letter codes.To: resv 3,5-OH; B: resv 4′-OH; H: DPPC phosphate group; Ta: DPPC ester group. For example, ToH: H-bonds between resv 3,5-OH and lipid phosphate oxygen; ToTa: H-bonds between Resv 3,5-OH and lipid ester group; ToTaH: H-bonds between resv 3,5-OH and both lipid phosphate group and ester group; BH: Hbonds between resv 4′-OH and lipid phosphate group.

We also observed that a specific z-position and a relatively flexible angle are both required to form a hydrogen bond with ester groups, therefore maximizing the resveratrol protection of the ester bond. Hydrogen bonding analysis was performed for all 18 resveratrol molecules during the last 90 ns trajectories. The percentage of each hydrogen bonding type between resveratrol and lipid are summarized in the histogram (Fig. 8). Among various types of hydrogen bonds, the majority of the resveratrol molecules have the double hydroxylated aromatic ring pointing towards the hydrophilic headgroups, forming a hydrogen bond with the ester group or the phosphate group of DPPC. The number of hydrogen bonds between the resveratrol and the lipids varies from 0 to 4. Among those hydrogen bonds, 46.6% are with ester group (carbonyl oxygen) of DPPC and 53.4% are with phosphate oxygen atoms on the head groups. On average, each resveratrol molecule forms on average 1.6 hydrogen bonds with two neighboring DPPC lipids, which explains the slightly tilted angles are more energetically favorable. The total number of hydrogen bonds formed between resveratrol and *sn2* chain ester group is slightly higher (1.2 times) than with *sn1* chain ester group.

### Resveratrol’s membrane protective effect from PLA2 and PLA1 hydrolytic attack

**Figure 9:**
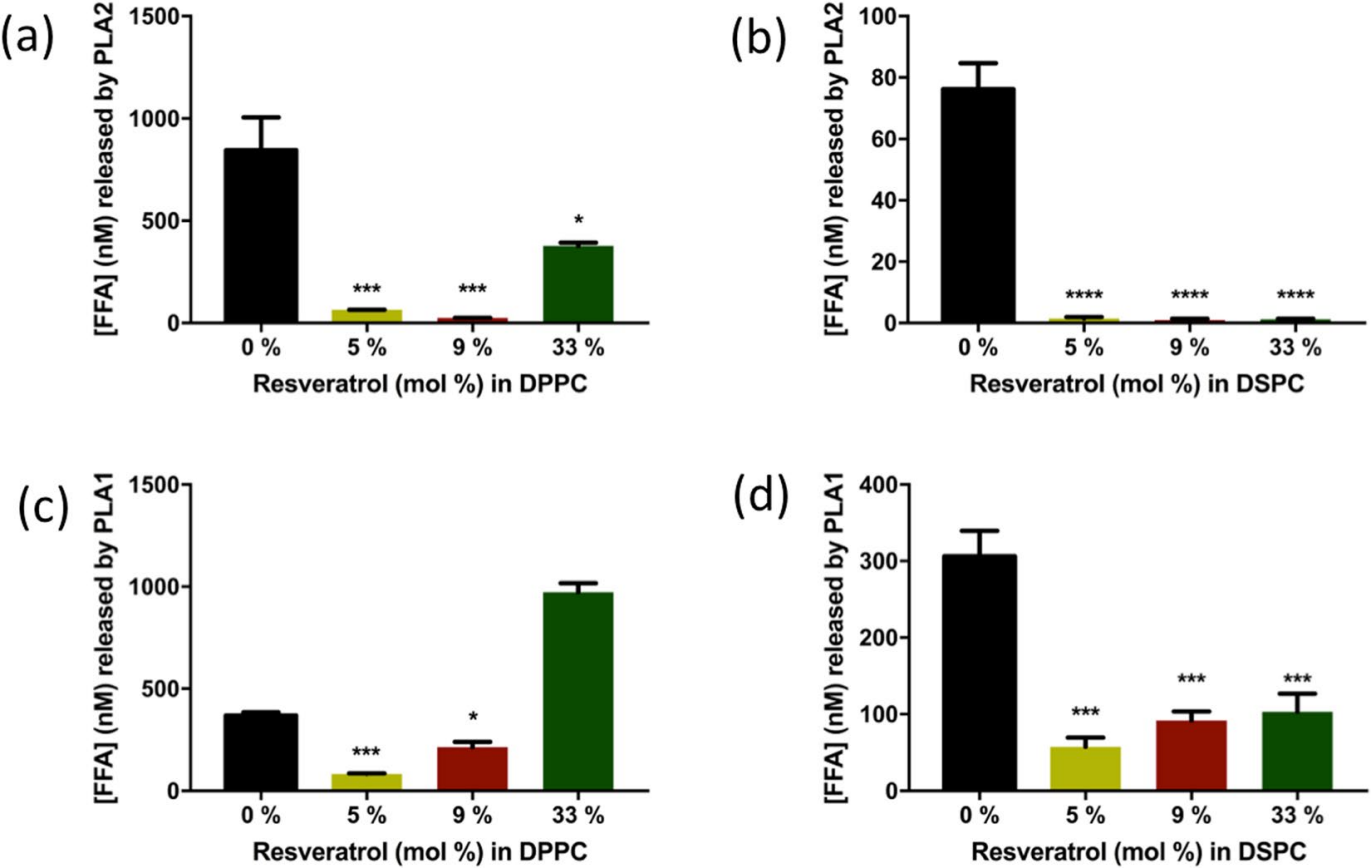
Resveratrol’s protective effect on DPPC and DSPC membranes from the hydrolytic activity of PLA2 or PLA1 at room temperature: Protection of resveratrol from PLA 2 on DPPC (**a**); or DSPC membranes (**b**). Protection of resveratrol from PLA 1 on DPPC (**c**); or DSPC membranes (**d**). Data are presented as free fatty acid (FFA) vs. DPPC or DSPC lipid membranes with different percentages of resveratrol. Results were analyzed using one-way ANOVA followed by Tuckey’s multiple comparison tests. The values are mean values ± the standard error of the mean (n = 3). Bars with asterisks denote significant difference from control (lipid membranes without resveratrol) (****p < 0.0001, ***p < 0.0006, *p < 0.02).

We hypothesized that the direct interaction observed between resveratrol and both *sn-*1 and *sn-2* ester groups of the phospholipid membranes from molecular dynamics simulations will prevent lipid ester hydrolysis. In other words, resveratrol may show a protective effect against PLA2 or PLA1 which attack and hydrolyze the *sn-*2, or *sn-1* ester bond of phospholipids, respectively, releasing a free fatty acid (FFA) and a lysophospholipid. We tested this hypothesis by quantifying the free fatty acid released when DPPC or DSPC membranes were challenged with PLA2 or PLA1 in the presence of 5, 9, 23 and 33% resveratrol. The hydrolysis of membrane phospholipids was monitored using Acrylodan-labelled Intestinal Fatty Acid Labelled Protein (ADIFAB) at room temperature. When ADIFAB binds to free fatty acids its fluorescence emission shifts^44,45^. In the presence of resveratrol the amount of free fatty acid released from the hydrolysis of DPPC and DSPC phospholipids was significantly lower compared to the control, which did not contain resveratrol. Therefore both DPPC and DSPC membranes in the presence of resveratrol were protected from the hydrolytic attack of PLA2 (Fig. 9a,b). We also observed membrane protection at 33% resveratrol in DPPC but less protection was achieved than when resveratrol was 5% or 9%. In the presence of PLA1, at 5 and 9 mole % resveratrol, DPPC membranes were protected but not at 33% (Fig. 9c). Also, DSPC membranes were protected from the hydrolytic activity of PLA1 at all resveratrol concentrations tested (Fig. 9d). The fact that 33% resveratrol offers less protection to DPPC membranes from PLA2 (Fig. 9a) or no protection from PLA1 (Fig. 9c), may due to fact that such high concentration of resveratrol causes the DPPC bilayer to be softer and thinner significantly, with a larger area per phospholipid having more chances to expose the *sn*-2 or *sn*-1 ester bonds away from the protective shield of resveratrol.

## Conclusion

**Figure 10:**
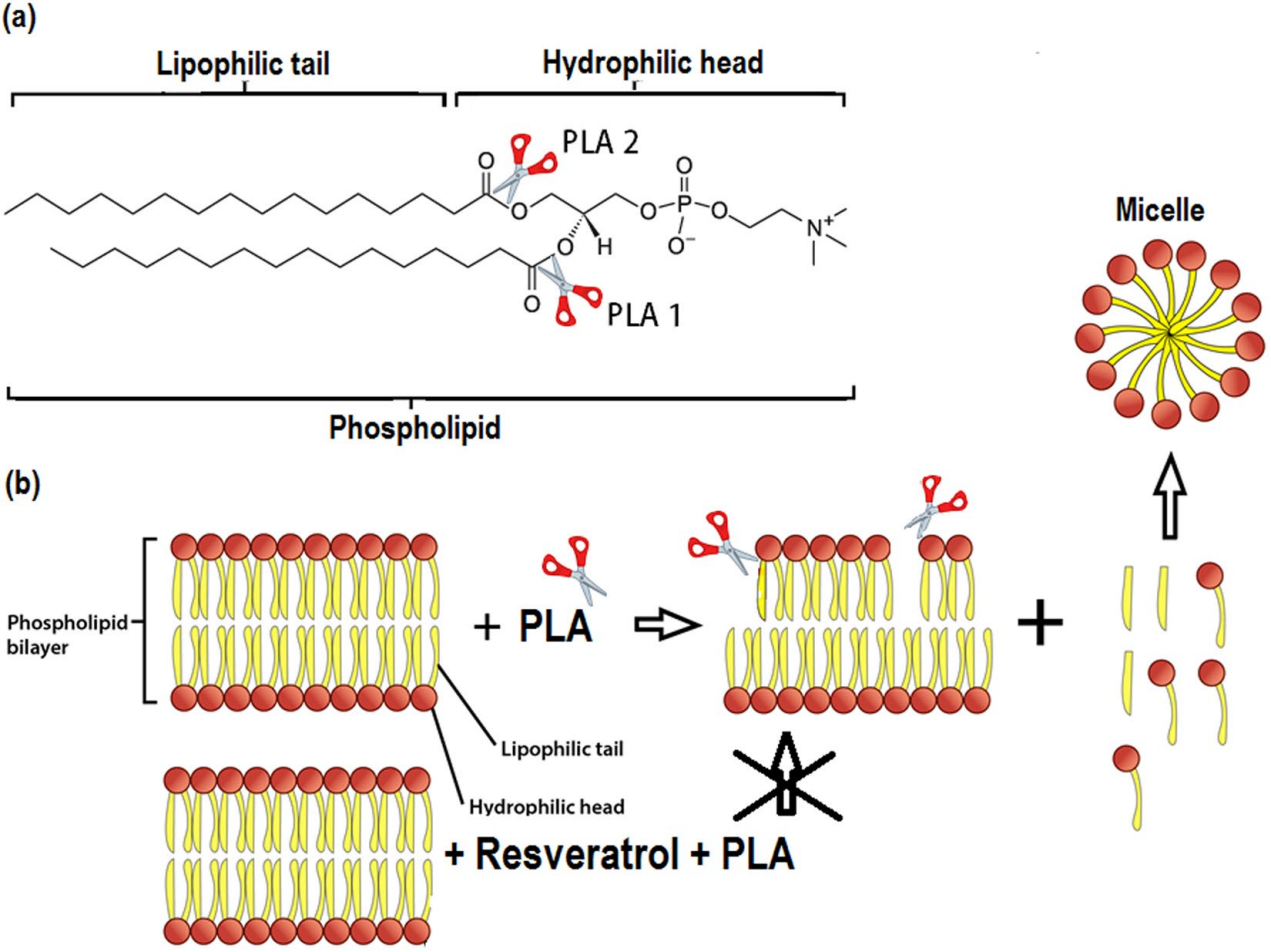
Mechanism of resveratrol protects lipid from PLA1 and PLA2. (**a**) Hydrolytic attack by PLA1 or PLA2 (red scissors) on the *sn*-1 or *sn*-2 ester bond of a phospholipid molecule, respectively. (**b**) Phospholipid bilayer membranes attacked by PLAs (1 or 2) release free fatty acid (yellow bars) and lysolipid molecules (yellow bars with red dots). Lysolipids then form micelles. In the presence of resveratrol, PLAs (1 or 2) phospholipid membranes are protected at most concentrations tested. Figure (**b**) is modified from Phospholipid Bilayer by OpenStax (used under CC BY 4.0).

In this study, we used computational and experimental approaches to reveal the molecular basis of the interaction between resveratrol and lipid membranes. Resveratrol molecules locate preferably near the interface of the hydrophilic head group and the hydrophobic fatty acid chain with a broad range of angular distribution. The majority of the resveratrol molecules have the double hydroxylated aromatic ring pointing towards the hydrophilic headgroup and forming one or two hydrogen bonds with the ester groups or the phosphate groups of the DPPC lipid. We further confirmed with PLA2 and PLA1 assays that this energetically favorable interaction between resveratrol and ester groups protects lipids from hydrolysis catalyzed by phospholipase at most concentration tested (Fig. 10). This novel mechanism described here can be used to explain various health benefits of resveratrol consumption. The details of resveratrol distribution and resveratrol-membrane interactions provide a new paradigm in designing more potent resveratrol analogs for cardiovascular protection.

## Methods

### Molecular dynamics simulations

Computational molecular dynamics simulations were performed to evaluate the interactions of DPPC and resveratrol. All MD simulations were run using the CHARMM36 (C36) lipid force field^30^. The TIP3P water model^46^ was used and CHARMM general force fields (CGenFF)^47^ for the resveratrol molecule. *Membrane Builder* in CHARMM-GUI was used to construct the solvated bilayer system^48–51^. 16 resveratrol molecules were placed randomly in the lipid bilayer of 80 DPPC per leaflet and 2 additional resveratrol molecules were placed in the bulk region. The system contained a ratio of 40 for water to lipid and a ratio of 8.8 for lipid to resveratrol. *Molecular Operating Environment* (MOE)^52^ was used to place 18 resveratrol molecules at random location and orientation. The bilayer systems were first equilibrated using the standard *Membrane Builder* six-step equilibration process and then 300 ns production runs were conducted at different temperatures.

All systems were simulated with a 2 fs time-step with NAMD version 2.9^53^. Long-range electrostatics was obtained by using the particle-mesh Ewald (PME) method^54^ with an interpolation order of 6 and a direct space tolerance of^6–10^. The van der Waals interaction was switched off from 10 to 12 Å by the force-based switching function^55^. Periodic boundary conditions were used for all simulations in the isobaric-isothermal (NPT) ensemble using Langevin thermostat and Andersen-Hoover barostat^56,57^. The pressure and temperature were maintained at 1 atm and 310 K or 325 K, respectively^56^. The analysis was performed using CPPTRAJ^58^, R^59^, and VMD program^60^. Surface area per lipid, bilayer thickness and deuterium order parameters were calculated using MEMBPLUGIN 1.1^61^.

### Materials and equipment

The following phospholipids were purchased from Avanti Polar Lipids (Alabaster, Alabama): Dipalmitoyl-phosphatidylcholine (DPPC) and Distearoyl-phosphatidylcholine (DSPC). *Trans*-Resveratrol (>99% purity) was obtained from TCI of America, Phosphate-Buffered Saline (PBS), pH 7.4 was purchased from Sigma-Aldrich, St. Louis, Missouri. Phospholipase A2 (PLA2) from honey bees, and PLA1 from *Thermomyces lanuginosus* were purchased from Sigma-Aldrich. Acrylodan-labelled Intestinal Fatty Acid Binding Protein (ADIFAB) and ADIFAB2 were purchased from FFA Sciences, San Diego, California.

#### Fluorescence measurement

The fluorescence intensities were measured and collected using CLARIOstar fluorimeter equipped with a plate reader from BMG Labtech. For ADIFAB2 the excitation wavelength was set at 375 nm and the emission wavelengths were set to 457 nm in the absence of FFA and 550 nm in the presence of FFA. For ADIFAB the excitation wavelength was set to 386 nm, the emission 432 nm in the absence of FFA and 505 nm in the presence of FFA.

### Preparation of liposomes

Multilamellar liposomes were prepared using DPPC or DSPC phospholipids. Mixtures containing 0, 5, 9, and 33 mole % resveratrol in DPPC or DSPC were dissolved in chloroform/methanol mixtures (2:1, v/v) in a 50-mL round bottom flasks. A dry lipid thin film was formed by evaporating the organic solvent using a rotary-evaporator. The lipid thin film was then hydrated with PBS solution at a temperature above the Tm of the phospholipid and vortexed and sonicated for 20 minutes resulting in multilamellar liposomes. Also, multilamellar liposomes without resveratrol were prepared and used as a control. All samples were prepared in triplicate.

### Differential Scanning Calorimetry (DSC)

DPPC or DSPC multilamellar liposomes containing 0. 9, 23, and 33 mole % resveratrol were prepared as described in the above section. The resultant dry lipid films were mixed with an amount of PBS thrice the amount of the dry lipid formulation and left to anneal overnight. A quantity of 3–5 mg of the hydrated sample was placed in crucibles and sealed. The samples were scanned using a Mettler 823 calorimeter at a scanning rate of 5 °C/min. Each formulation had three independent replicates and each replicate was scanned at least three times until identical scans were obtained.

### Measurement of FFA after PLA hydrolytic attack

The stability/integrity of lipid membranes in the presence of different concentrations of resveratrol was challenged with PLA2 at room temperature. PLA1 hydrolyzes the *sn*-1 whereas PLA2 hydrolyses the *sn*-2 ester bond of phospholipids and in both cases free fatty acid (FFA) and a lysolipid are released. The FFA produced by PLA1 (Lipid:PLA1, 12.5 mg:4 ul. The quantity of 4ul of PLA1 represents >0.048 KLU) was measured using ADIFAB kit according to the instructions of the manufacturer. The FFA produced by PLA2 was measured using ADIFAB 2 kit for sensitivity purposes. Here we give a short description as to how we performed the ADIFAB2 measurements. Briefly, a quantity of 1 ml of different formulations of multilamellar DPPC or DSPC liposomes containing 0, 5, 9 or 33 mole % resveratrol was mixed with 0.005 mg of PLA2. There was 12.5 mg of phospholipid per ml of phospholipid formulation. The mixture of phospholipid formulations with PLA2 was left to react for 10 min and then centrifuged. Furthermore, liposome formulations without PLA2 were used as a background control. The concentration of unbound FFA was determined by the ratio of fluorescence at 550 nm and 457 nm. 0.5 uM of ADIFAB2 was added to each well in a 96-well black plate containing the sample (4% of total volume) and measuring buffer (50 mM HEPES, 140 mM NaCl, 5 mM KCl, 1 mM Na_2_HPO_4_). 24 µM of Bovine Serum Albumin (BSA) in measuring buffer was served as the control to determine the ratio of ADIFAB2 in the absence of FFA (R_0_). The fluorescence intensity was measured using CLARIOstar fluorometer at excitation wavelength 375 nm and reading at wavelengths 550 nm and 457 nm. Measurements of the blank wells (HEPES buffer and samples) before adding ADIFAB2 were also taken. The analysis was carried out in triplicate. The concentration of FFA was calculated using the following equations:

1 $$Ro=\frac{{I}_{550\,}^{o}-{I}_{550\,}^{blank}}{{I}_{457\,}^{o}-{I}_{457\,}^{blank}}$$

2 $$R=\frac{{I}_{550}-{I}_{550\,}^{blank}}{{I}_{457}-{I}_{457\,}^{blank}}$$

3 $$[FFA]={K}_{d}\times Q\times \frac{R-{R}_{o}}{{R}_{max}-R}$$

4 $$R=(\frac{{R}_{0measured}}{{R}_{ocalibration}})\times {R}_{\max calibration}$$

Where R_o_ is the ratio of ADIFAB2 in HEPES measuring buffer in the absence of FFA, R is the ratio of ADIFAB2 in the presence of membranes with or without PLA. The constants K_d_, Q and R_max calibration_ were determined and provided by FFA Sciences (K_d_ = 32, Q = 5, R_max calibration_ = 0.762). The Fig. 6 graphs report the [FFA] calculated as follows:

5 $$[{\rm{FFA}}]={[{\rm{FFA}}]}_{{\rm{PLA}}}-{[{\rm{FFA}}]}_{{\rm{background}}}$$

where_,_ [FFA]_background_, Is the background calculated in the presence of ADIFAB, and phospholipids with resveratrol only (without PLA), and [FFA]_PLA_ is the free fatty acid concentration calculated after membranes with resveratrol reacted in the presence of PLAs.

### **D**ata Availability

All relevant data are available from the authors on request and/or are included with the manuscript. The R scripts and pytraj scripts used to generate the free energy plots can be found on GitHub https://github.com/LynaLuo-Lab/Membrane-Resveratrol.
